# Inertia Controlled Capillary Pressure at the Juncture between Converging and Uniform Channels

**DOI:** 10.1038/s41598-019-49588-x

**Published:** 2019-09-25

**Authors:** Harris Sajjad Rabbani, Thomas Daniel Seers

**Affiliations:** Department of Petroleum Engineering, Texas A&M University at Qatar, Education City, Doha Qatar

**Keywords:** Hydrology, Fluid dynamics

## Abstract

In this research, we reveal the transient behavior of capillary pressure as the fluid-fluid interface travels across the juncture between a converging and uniform capillary, via high-resolution CFD (Computational Fluid Dynamics) simulations. Simulations were performed at different wetting conditions (strong-wet and intermediate-wet) and capillary wall convergence angles. Our results demonstrate that as the angle of convergence increases, capillary pressure at the junction decreases commensurately. Moreover, in contrast to strong-wet conditions, the profile of capillary pressure at the converging-uniform capillary juncture under intermediate-wet conditions is highly non-monotonic, being characterized by a parabola-like form. This non-monotonic behavior is a manifestation of strong inertial forces governing dynamic fluid-fluid interface morphology. This yields conditions that promote the advancement of the fluid-fluid interface, as inertial forces partially nullify the capillary pressure required for the immiscible interface to enter the uniform capillary. In addition to numerical analysis detailed above, a novel theoretical stability criteria that is capable of distinguishing between stable (capillary dominated) and unstable (inertia dominated) interfacial regimes at the converging-uniform capillary juncture is also proposed. In summary, this fundamental study offers new insights into the interface invasion protocol, and paves the way for the re-evaluation of capillary junction controlled interfacial dynamics.

## Introduction

Defined as the pressure difference across a fluid-fluid interface, capillary pressure is a first-order control over immiscible fluid displacement within numerous porous materials, and is fundamental to the study of the multiphase flow central to a wide variety of natural and industrial processes such as cardiac transcapillary exchange^1^, hydrocarbon migration and production^2^, geological sequestration of carbon dioxide^3^, unsaturated flow through the vadose zone^4^, water transport within hydrogen fuel cells^5^ and drying mechanics^6^. Mason and Morrow^7^ performed a theoretical study to highlight the effect of cross-sectional angularity of channel on the capillary pressure behavior. They demonstrated that under perfectly-wet conditions the capillary pressure required for the interface to displace the residing fluid increases as the angularity of channel enhances. Later Ma *et al*.^8^ used the Mayer and Stowe-Princen^9^ (MS-P) theory of drainage to investigated the behavior of interface in angular capillaries under partial-wet conditions. They revealed instability in capillary pressure profile under partial-wet conditions, which was associated with the complex relationship between the channel angularity and the contact angle between the fluid-fluid interface and solid surface. While the focus of the majority of previous work has been on the uniform capillary, some studies^10,11^ have also investigated the behavior of interface in a capillary channel with non-uniform cross-section. Rabbani *et al*.^10^ performed CFD simulation to examine the capillarity of the converging-diverging channel, illustrating that under intermediate-wet conditions there is a shift in the direction of capillary forces as interface traverses from converging to uniform capillary that consequently results in a change in displacement mechanism from drainage to imbibition. Although the underlying physics and theoretical tools to quantify capillary pressure inside uniform capillaries^7–9,12^ and capillaries with axial variation (converging or diverging capillary)^10,11^ are well known, the behavior of capillary pressure at the juncture between capillary channels of discordant orientation, and fundamental principles controlling such phenomena have, hitherto, remained elusive. Given the commonly disparate and disordered geometry of many natural and manufactured pore networks, the behavior of capillary pressure at the capillary junction merits further investigation.

The objective of this work is to delineate the behavior of capillary pressure at the juncture between converging and uniform capillaries under dynamic conditions. To achieve this aim, we performed high-resolution two-phase flow simulations through converging-uniform capillaries using computational fluid dynamics (CFD) based modelling at various wetting conditions and capillary wall orientation angles (i.e. angles of convergence). The capillary pressure profile was computed as the fluid-fluid interface enters from converging to uniform capillary. The invasion protocol within this study is limited to drainage conditions (invading fluid is non-wetting) only.

## Practical Applications

**Figure 1:**
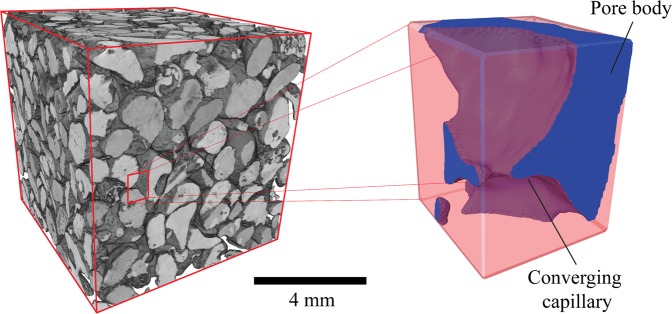
Modern bioclastic carbonate sand (Fuwairit Beach, NE Qatar) imaged within Thermo-Fisher Heliscan x-ray micro-computed tomography scanner at a resolution of ~3.5 µm. A subvolume is extracted from the sand pack to illustrate the geometry of converging capillaries that typically connect pore bodies to pore throats within particulate based porous media.

Porous media composed primarily of packed particulate (e.g. unconsolidated sediments and soils, sedimentary rocks, sand bed filters, ceramic coatings, catalyst particle beds etc.) typically contain pore networks comprising of intergranular pore bodies connected to comparatively smaller pore throats formed between adjacent grain contacts via converging capillary channels (Fig. 1).

Textural disparities in both natural and synthetic particulate columns, owing to variations in grain size, grain shape, sorting and packing configuration, give rise to intrinsic variability in the angles of convergence between interconnected pore bodies and pore throats (see Fig. 1). Despite the ubiquity of converging capillary geometries within many naturally occurring and engineered porous media, there is still a lack of understanding over how the relative rate of convergence along connecting capillaries impacts upon immiscible pore invasion, particularly at the juncture between the pore throat and converging capillary channel. Motivated by the above, this work seeks to investigate fluid-fluid interfacial mechanics at the junction between converging-uniform capillary. We propose that this simplified model can provide an improved understanding of multiphase fluid flow in through capillary junctures inhabiting a diverse array of porous media. The knowledge gained from this fundamental study can further improve the theoretical models employed in forecasting the fluid flow profiles in geological as well as synthetic porous media.

It is known from the literature that the efficiency of immiscible displacement enhances as the wetting conditions of porous media changes from strong to intermediate^10,13–15^. Our study rationalizes the underlying physics of such phenomena indicating that the synergistic impact of increase in convergence angle and contact angle dampens the capillary resistive forces acting along the interface, further promoting the fluid invasion in porous medium. This insight is potentially transformative towards for reservoir engineering, geoengineering and hydrogeology/soil science applications (i.e. oil and gas accumulation and production, the geological sequestration of CO2, methane emission within the gas-hydrate/free gas transition zone, fluid flow within the vadose zone), as well for chemical engineers in managing water flooding issues in fuel cells. In addition to aforementioned porous media, an understanding of the immiscible fluid flow characteristics at capillary junctures also play a key role in the design and optimization of microfluidic devices (e.g. droplet generators).

### Computational fluid dynamics simulations

We performed Computational Fluid Dynamics (CFD) simulations using C++ library OpenFOAM (Open source Field Operation And Manipulation). InterFoam: a solver of OpenFoam, was utilized to compute the Navier-Stokes equations which are coupled with the volume of fluid method to track the fluid-fluid interface. The details of the numerical formulation are described elsewhere in detail^15^, and therefore will not be repeated here.

**Figure 2:**
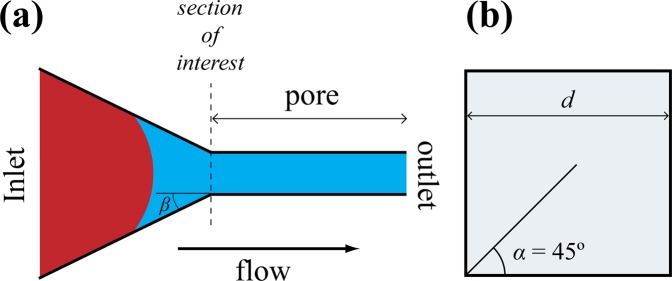
The geometrical model used to investigate the dynamic behavior of capillary pressure at the junction of converging-uniform capillary. (**a**) Schematic showing plane view of the model. The inlet supplies the invading fluid at constant flow rate. The section of interest is the junction between converging and uniform capillary. The outlet is equalized to constant atmospheric pressure. The direction of flow is from left to right. (**b**) Represents the cross-section of uniform capillary which is square with half-corner angle α = 45° and diameter d.

The flow domain was 3D in nature that consisted of a converging capillary which is further connected to a uniform capillary of diameter *d* at an angle of orientation *β* (Fig. 2a).

The relationship between *β* and *d* can be mathematically described as;

1 $$\tan \,\beta =\frac{D-d}{2c}$$

where *D* is the inlet diameter and *c* is the distance between uniform capillary and the inlet. The 3D view of complete geometry is shown in Fig. S1 of the Supplemental Information.

The cross-section of both capillaries is square with half corner angle *α* value of 45° (Fig. 2b). Initially, the uniform capillary is completely filled by defending fluid (viscosity ${\mu }_{d}=1.0\times {10}^{-3}$ Pa·s and density ${\rho }_{d}=1000$ kg·m^−3^) and the fluid-fluid interface resides in the converging channel close to the junction. The inlet of converging capillary supplies the invading fluid (viscosity ${\mu }_{i}=1.0\times {10}^{-3}$ Pa·s and density ${\rho }_{i}=1000$ kg·m^−3^) for the displacement at a constant flow rate $Q=7.0\times {10}^{-11}$ m^−3^·s^−1^, while the outlet of the uniform capillary is maintained at atmospheric pressure. The interfacial tension between the fluids is *σ* = 70 × 10^−3^ N·m^−1^. The static contact angle *θ*, which corresponds to the angle between the defending fluid and solid surface is recognized as the direct measure of the wettability of system and was included as input parameter in the solver.

Series of 3D numerical simulations were performed with *β* = 21.8°, 38.7° and 50.2°. The *β* values were varied by changing the diameter of uniform capillary *d* as 0.8 × 10^−3^ m, 0.6 × 10^−3^ m and 0.4 × 10^−3^ m in accordance to Eq. 1. Two different *θ* values 30° (strong-wet) and 60° (Intermediate-wet) were considered in this research. As the simulation begins, the fluid-fluid interface traverses from the converging capillary to the uniform capillary.

Our simulations do not take into account contact angle hysteresis and dynamic contact angle effects. Moreover, since the fluid densities are identical, gravity forces do not play a role in the immiscible displacement. Visualization and post-processing of the numerical results were performed using ParaView^16^.

At each time step the saturation of defending (wetting) fluid *s*_*w*_ at the cross-section of the junction of converging-uniform capillary (see Fig. S2 in the Supplemental Information) was determined, and subsequently the capillary pressure *p*_*c*_ was computed by the Laplace equation

2 $${p}_{c}=\sigma k$$

where *k* m^−1^ is the curvature of fluid-fluid interface, estimated using the approach of Rabbani *et al*.^15^.

## Results and Discussion

### Capillary pressure at the converging-uniform capillary juncture

**Figure 3:**
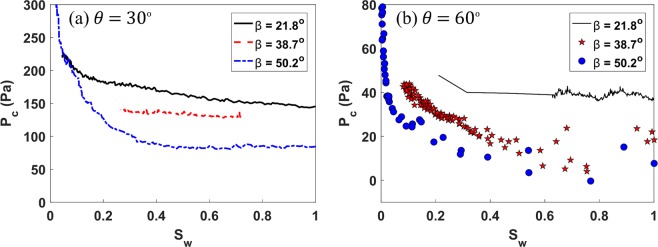
Capillary pressure *p*_*c*_ against saturation of defending fluid *s*_*w*_ at various *β* values under (**a**) strong-wet conditions (*θ* = 30°) and (**b**) intermediate-wet conditions (*θ* = 60°) as the fluid-fluid interfaces traverses the converging-uniform capillary juncture. For both wetting conditions *p*_*c*_ decreases as the *β* increases (decrease in the size of uniform capillary *d*), which is contrary to conventional Young-Laplace law of uniform capillary which necessitates an increase in *p*_*c*_ with a corresponding decrease in *d*. The reader should also note the marked contrast in capillary pressure profiles between the m°notonic trend for *θ* = 30° and the comparatively noisy, quasi-parabolic *p*_*c*_ trend for *β* = 38.7° and 50.2° under intermediate-wet conditions (*θ* = 60°), indicating pronounced instabilities in the fluid-fluid interface.

The behavior of capillary pressure *p*_*c*_ as a function of defending fluid saturation *s*_*w*_ under strong-wet conditions (*θ* = 30°) and intermediate-wet conditions (*θ* = 60°) is displayed in Fig. 3. In the case of both of the studied wetting conditions, *p*_*c*_ decreases with increasing *β* (and thus decline in size of the uniform capillary *d*), indicating reduction in the resistance offered by capillary forces, and therefore less energy required by the fluid-fluid interface to advance into the uniform capillary. The results presented in Fig. 3 are in direct opposition to the Young-Laplace Law of a uniform capillary, that defines the inverse relationship between *p*_*c*_ and *d*. It is important to note that we performed additional simulations where *β* was varied by changing the inlet diameter *D* while keeping the diameter of uniform capillary *d* constant, the results are shown in Fig. S3 of Supplemental Information. Overall the trend indicated by Fig. 3 is consistent with Fig. S3 elucidating that *p*_*c*_ at junction strongly depends upon the converging angle of the flow domain and the wettability that results in ${p}_{c}\propto \,\cos (\theta +\beta )$, as shown in Rabbani *et al*.^10^ which can also be ascertained from Fig. 3.

As the *θ* changes from 30° to 60°, the capillary pressure profile starts to display highly non-monotonic behavior (see Fig. 3b). Though small scale instabilities are present in the case of *β* = 21.8° at *θ* = 60°, overall the *p*_*c*_ curve displays smooth and regular trend, which is consistent with strong-wet conditions. This is in marked contrast to the case of *β* = 38.7° and 50.2° under equivalent wetting conditions, where the trend is highly non-monotonic, and subtly parabolic in form. Moreover, at *β* = 50.2°, owing to this non-monotonic behavior, there exists a temporary reversal in the displacement mechanism from drainage to imbibition. This enigmatic *p*_*c*_ behavior at the junction, manifested under intermediate-wet conditions when *β* = 38.7° and 50.2°, is highly novel and absent from current theories concerning pore-scale invasion protocol.

**Figure 4:**
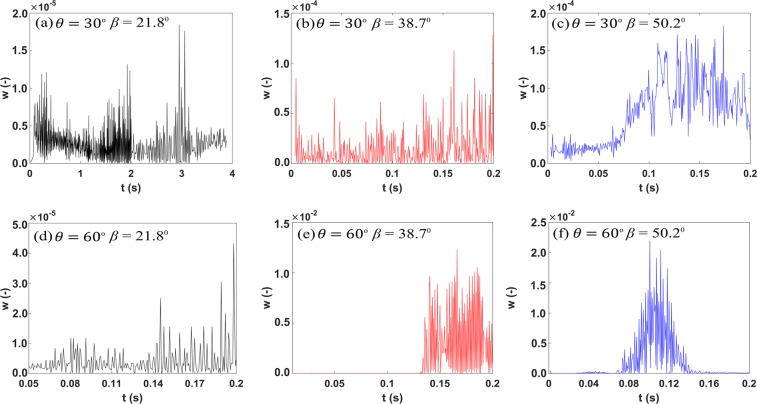
Variation in the Weber number *w*, characterized as $w=\frac{{\rho }_{i}{|\frac{{\rm{\partial }}l}{{\rm{\partial }}t}|}^{2}d}{\sigma \,\cos (\theta )}$ against time *t* (*s*) (**a**) under strong-wet conditions and (**b**) intermediate-wet conditions. In case of (**a–d**), *w* remains almost constant throughout the displacement process. However, in case of *β* = 38.7° and 50.2°, *w* is enhanced as the interface approaches the converging-uniform capillary junction, signifying the strong influence of inertial forces over fluid-fluid interface morphology, resulting in the non-monotonic *p*_*c*_ behavior displayed in Fig. 3b.

To further characterize the *p*_*c*_ behavior of the fluid-fluid interface at the converging-uniform capillary juncture, we have mapped associated variations in the Weber number *w*, which defines the relative importance of inertial forces over capillary forces, as the interface moves along the junction of converging-uniform capillary (Fig. 4). Traditionally as shown in previous studies^17,18^ *w* is expressed as;

3 $$w=\frac{{\rho }_{i}{|\frac{\partial l}{\partial t}|}^{2}d}{\sigma \,\cos (\theta )}$$

where *l* and *t* represents the distance travelled by the interface and time it require to cover *l* respectively.

It is apparent from Fig. 4 that under intermediate-wet conditions (*θ* = 60°) at *β* = 38.7° and 50.2°, *w* displays marked enhancement as the fluid-fluid interface enters the converging-straight capillary juncture: behavior that is notably absent from other simulated cases. It is clear from Fig. 4 that as the orientation of junction and *θ* increases, inertial effects on fluid-fluid interfacial dynamics are enhanced. This in turn results in a decline in *p*_*c*_, which ultimately engenders the non-monotonic (parabolic) form of the *p*_*c*_ trend displayed in Fig. 3b.

Though it is known from the literature that inertia plays a prominent role in governing the motion of interface^19–21^, the exact nature of how inertia contributes towards dynamic interface behavior have remained, hitherto, unresolved. Our numerical results unravel this phenomena, rationalizing the underlying physics. It can be clearly seen from Figs 3b and 4e,f that even under quasi-static conditions, curvature of the interface at the capillary juncture is a function of inertial force. As the mass transportation of invading fluid across the junction increases (decrease in *s*_*w*_), the relative influence of inertial forces is enhanced (increase in *w*), which in turn, promotes the reduction in curvature of fluid-fluid interface decreasing the *p*_*c*_ required to enter the channel. Thus, the narrower the juncture is relative to the converging capillary, the larger the influence of inertia at the juncture will be, and the greater ease with which the fluid-fluid interface will enter the capillary.

Figures 3 and 4 clearly highlight the importance of the geometric configuration of the junction in controlling the pore-scale interface behavior, and demonstrate the mediating effect this can have on the stability of macroscopic immiscible fluid displacement. Moreover, we have established that under intermediate-wet conditions, the morphological characteristics of junction can have a profound impact upon pore-scale invasion events. Therefore, we suggest that current pore network models^22,23^ that are based on pore filling rules proposed by Lenormand *et al*.^24^ may require revision to incorporate the effect of the capillary junction on invasion protocols. Moreover, inertial forces are commonly ignored in pore network modelling approaches^25^. Our results suggest that this can be a reasonable assumption for strong-wet conditions, but under intermediate wet conditions the localized impact of inertia on interface invasion is important to capture, as demonstrated in Figs 3b and 4e,f.

### Stability criterion

In this section we will be proposing a new modified Weber number: w^*^, that can be used as a diagnostic tool to distinguish between an inertia controlled interfacial regime and a capillary dominated interfacial regime at the juncture between a converging-uniform capillary channel. The new modified Weber number w^*^ is shown as Eq. 4 below.

4 $${w}^{\ast }=\frac{\frac{2{\rho }_{i}\frac{\partial (\frac{\partial l}{\partial t})}{\partial t}[2d+cos(\beta )\frac{[D-d]}{2}]}{\cos (\theta )tan(\beta )}-\frac{2\sigma \,\cos (\theta +\alpha )}{d}}{[\frac{\pi \sigma (1-\frac{\beta }{\pi })\sin (\pi -2\beta )}{\sin (\beta )}][\frac{\cos (\theta +\beta )+cos\theta }{d}]}$$

**Figure 5:**
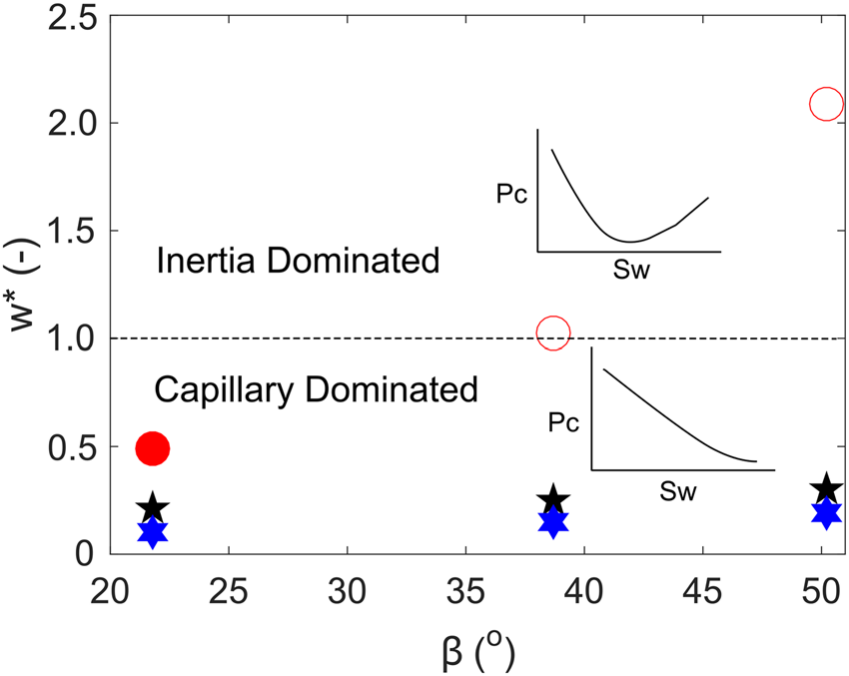
Average value of modified Weber number $|{\bar{w}}^{\ast }|$ for the simulations presented in Figs 3 and 4 against β for *θ* = 0° (Hexagram p°ints), 30° (Star points) and 60° (Circle points). $|{\bar{w}}^{\ast }|$ = 1 (dashed line) is criteria for distinguishing between inertia dominated regime ($|{\bar{w}}^{\ast }|$ > 1) and capillary dominated regime ($|{\bar{w}}^{\ast }|$ < 1). Filled points shows simulation cases where capillary dominated interfacial regime at the juncture, whereas open symbols represents instability at the juncture.

For an inertia controlled interfacial regime $|{w}^{\ast }|$ > 1, while for capillary dominated regime $|{w}^{\ast }|$ < 1. The details of the derivation of Eq. 4, are given in Supplementary Information. Figure 5 shows the modified weber number $|{\bar{w}}^{\ast }|$ averaged over *s*_*w*_ = 0 to *s*_*w*_ = 1 for each simulated case against *β*.

It can be clearly seen from Fig. 5 that under intermediate-wet conditions (*θ* = 60°), $|{\bar{w}}^{\ast }|$ > 1 for *β* = 38.7° and 50.2°, while for all other cases $|{\bar{w}}^{\ast }|$ < 1. Notably, Fig. 5 offers validation for the proposed modified Weber number w^*^ (Eq. 4), which can be calculated to gather insights into the *p*_*c*_ behavior at the junction. As indicated in previous section, under inertia dominated conditions, the *p*_*c*_ trend is highly non-monotonic, whereas strong capillary forces result in a highly monotonic *p*_*c*_ trend.

## Summary and Conclusion

In this research we performed CFD (Computational Fluid Dynamics) simulations with high spatial and temporal resolution to delineate capillary pressure behavior at the juncture between and converging and uniform capillary channel. The studied capillary model consisted of a converging capillary, connected to a uniform capillary via an obtuse junction, with the sense of displacement from the converging channel into the uniform capillary under drainage conditions. The simulations were conducted under different wetting conditions (strongly water-wet and intermediate wet) and angles of convergence, with capillary pressure computed as the fluid-fluid interface enters the junction.

It can be concluded from our results that as the orientation angle increases, capillary pressure required to enter the uniform capillary decreases. In addition, we have demonstrated that, in contrast to strong-wet conditions, the nature of capillary pressure curve under intermediate-conditions is highly non-monotonic, which is related to strong, localized inertial control on the interface morphology at the junction. In addition, we also proposed a novel theoretical model (stability criterion) that is capable of distinguishing between inertia and capillary dominated interfacial regimes at the junction of converging-uniform capillary.

## Supplementary information

Inertia Controlled Capillary Pressure at the Juncture between Converging and Uniform Channels

## Data Availability

The data presented in this manuscript will be available freely via sending a request to the corresponding author.
