# Supplementary material for: Inertia Controlled Capillary Pressure at the Juncture between Converging and Uniform Channels
Source: Sci Rep. 2019 Sep 25;9:13870. doi: 10.1038/s41598-019-49588-x (PMC6761262; doi:10.1038/s41598-019-49588-x)
Supplement: Supplementary file 1 — Inertia Controlled Capillary Pressure at the Juncture between Converging and Uniform Channels [file 41598_2019_49588_MOESM1_ESM.docx]

**Inertia Controlled Capillary Pressure at the Juncture between Converging and Uniform Channels**

Harris Sajjad Rabbani and Thomas Daniel Seers

Department of Petroleum Engineering, Texas A&M University at Qatar, Education City, Doha, Qatar

**Supplementary Information**


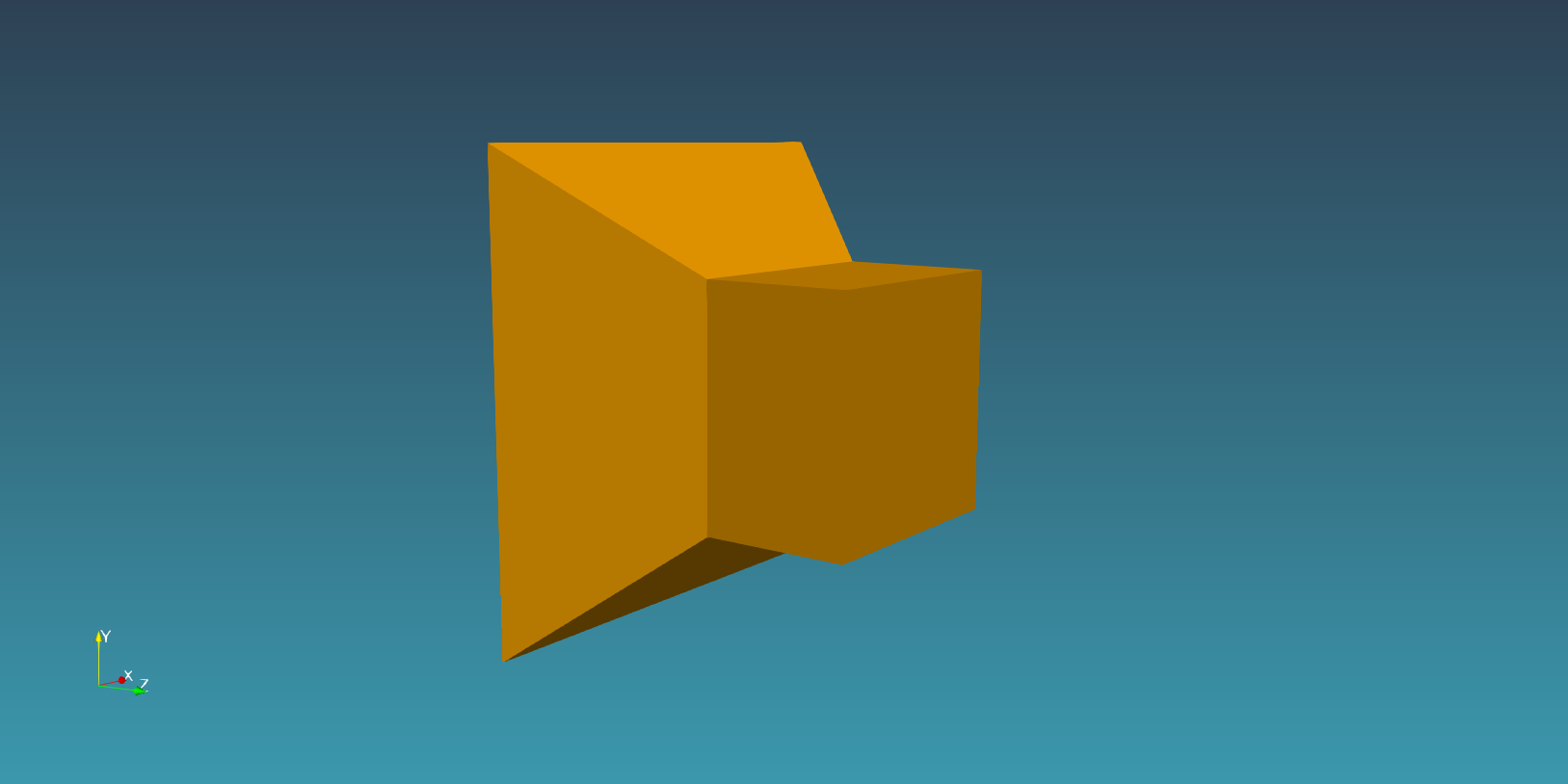


**Figure S1.** 3D view of the model.


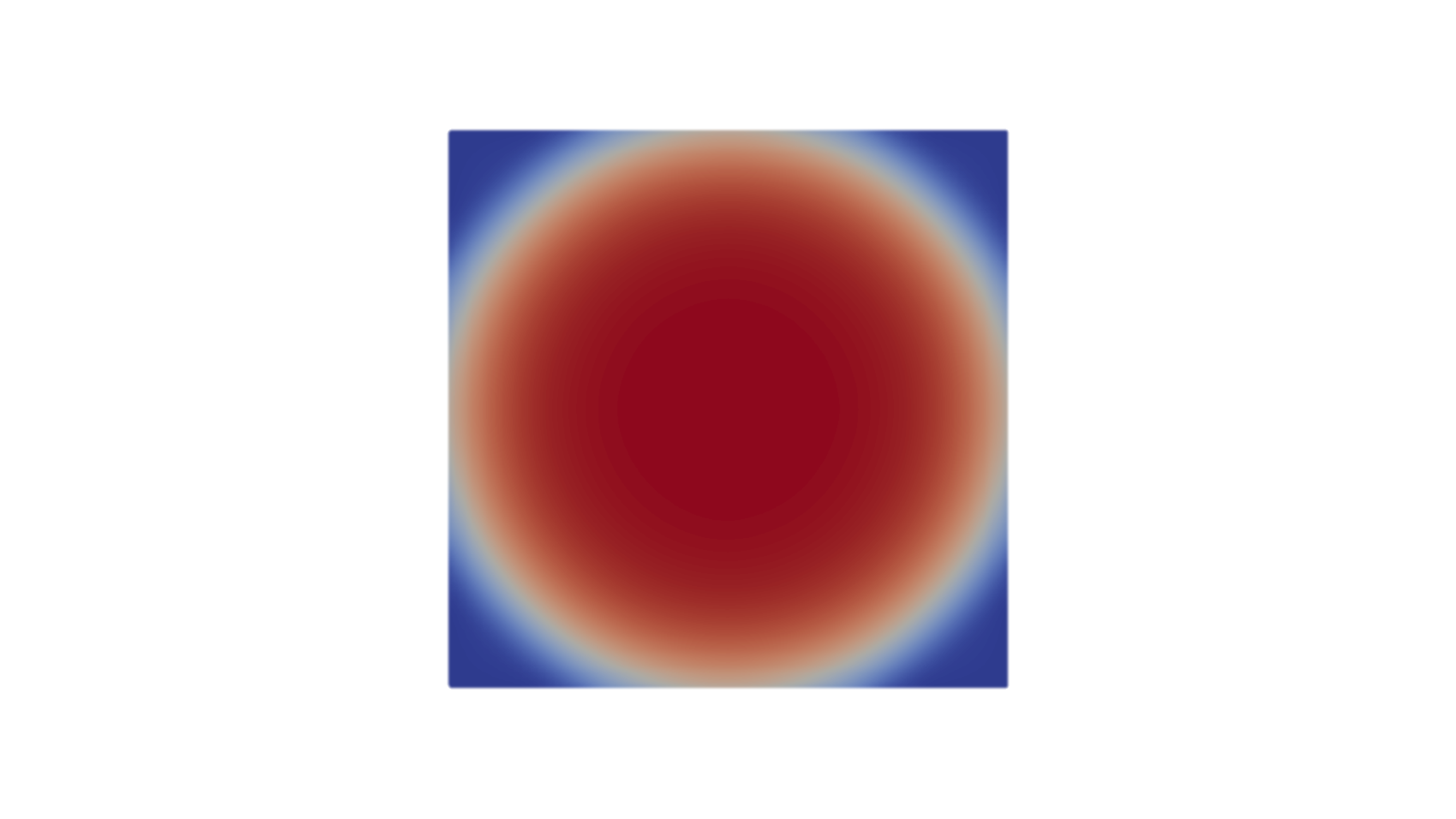


**Figure S2.** Cross-section of juncture showing the invading (non-wetting) fluid as red and defending (wetting) fluid as blue. Intermediate colors represents fluid-fluid interface.


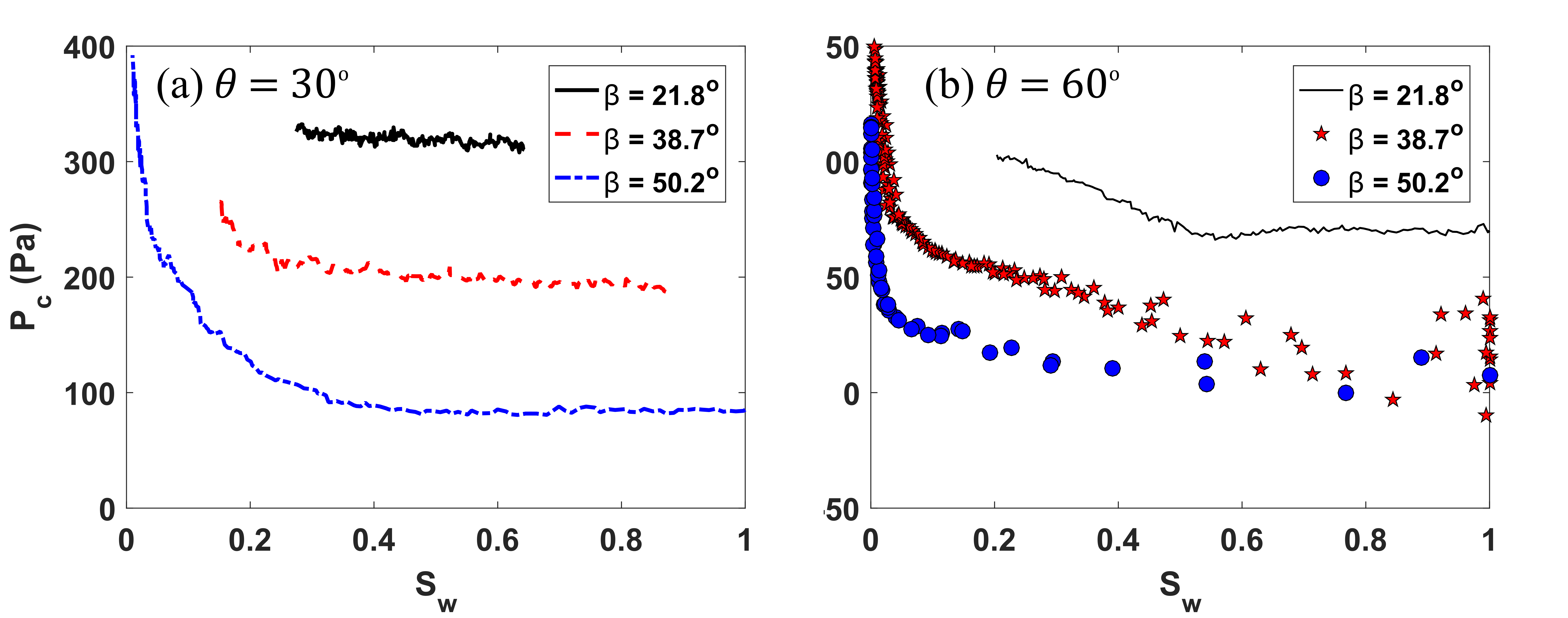


**Figure S3.** Capillary pressure $p_{c}$ against saturation of defending fluid $s_{w}$ at various angle of orientation $\beta$ under (a) strong-wet conditions ($\theta$ = 30^o^) and (b) intermediate-wet conditions ($\theta$ = 60^o^) as the fluid-fluid interfaces traverses the converging-uniform capillary juncture. In contrast to the results shown in the main text, for the results shown in Fig. S3 $\beta$ was varied by changing the inlet diameter $D$ while keeping the diameter of uniform capillary $d$ constant. These results are consistent with Fig. 3 indicating that rather than $D$ or $d$ it is $\beta$ and contact angle $\theta$ that control interfacial dynamics at the capillary juncture.

*Derivation of the Modified Weber Number*

We start with force balance at the cross-section of junction of converging-uniform capillary. The force balance per unit of interfacial area can be formulated as

$\frac{f_{i_{invading}}+ f_{i_{defending}}+ f_{i_{converging capillary}}}{cos(\theta)tan(\beta)}= f_{c_{converging capillary}}+ f_{c_{uniform capillary}}+ f_{c_{corner angle}}$ (1)

where $f_{i}$ and $f_{c}$ are the components of inertial and capillary force respectively. Substituting $f_{c_{uniform capillary}}$ with $\frac{\pi\sigma\left( 1-\frac{\beta}{\pi} \right)sin(\pi-2\beta)cos\theta}{\sin\left( \beta\right)d}$ : the equation proposed by Rabbani et al.^10^ for uniform capillary with square cross-section, representing $f_{c_{converging capillary}}$ with $\frac{\pi\sigma\left( 1-\frac{\beta}{\pi} \right)sin(\pi-2\beta)\cos\left( \theta+\beta\right)}{\sin\left( \beta\right)d}$ as given in Rabbani et al.^12^, expressing $f_{c_{corner angle}}$ as $\frac{2\sigma cos(\theta+\alpha)}{d}$, and representing $f_{i_{invading}}$, $f_{i_{defending}}$ and $f_{i_{converging capillary}}$ as $\rho_{i}\frac{\partial\left( \frac{\partial l}{\partial t} \right)}{\partial t}d$, $\rho_{d}\frac{\partial\left( \frac{\partial l}{\partial t} \right)}{\partial t}d$ and $\rho_{i}\frac{\partial\left( \frac{\partial l}{\partial t} \right)}{\partial t}\frac{D}{cos(\beta)}$ respectively yields

$\frac{\rho_{i}\left| \frac{\partial\left( \frac{\partial l}{\partial t} \right)}{\partial t} \right|\left[ 2d+ cos(\beta)\frac{[D-d]}{2} \right]}{cos(\theta)tan(\beta)}= \left[ \frac{\pi\sigma\left( 1-\frac{\beta}{\pi} \right)sin(\pi-2\beta)}{sin(\beta)} \right]\left[ \frac{\cos\left( \theta+\beta\right)+cos\theta}{d} \right]+ \frac{2\sigma cos(\theta+\alpha)}{d}$ (2)

In Eq. 2, $l$ represents the distance travelled by the interface, and $D$ is the length (i.e. diameter) of the inlet. The above equation can be further rearranged to describe a new form of Weber number: $w^{*}$ which can be used to define the interfacial regime at the capillary junction

$w^{*}= \frac{\frac{{2\rho}_{i}\frac{\partial\left( \frac{\partial l}{\partial t} \right)}{\partial t}\left[ 2d+cos(\beta)\frac{[D-d]}{2} \right]}{cos(\theta)tan(\beta)}-\frac{2\sigma cos(\theta+\alpha)}{d}}{\left[ \frac{\pi\sigma\left( 1-\frac{\beta}{\pi} \right)sin(\pi-2\beta)}{sin(\beta)} \right]\left[ \frac{\cos\left( \theta+\beta\right)+cos\theta}{d} \right]}$ (3)
